# Supplementary material for: An Economic Evaluation of ‘Sheds for Life’: A Community-Based Men’s Health Initiative for Men’s Sheds in Ireland
Source: Int J Environ Res Public Health. 2022 Feb 15;19(4):2204. doi: 10.3390/ijerph19042204 (PMC8871832; doi:10.3390/ijerph19042204)
Supplement: Supplementary file 1 [file ijerph-19-02204-s001.zip › ijerph-1535665-supplementary.pdf]

Supplementary File S1: Histograms of Six dimensions of the SF-6D at baseline.

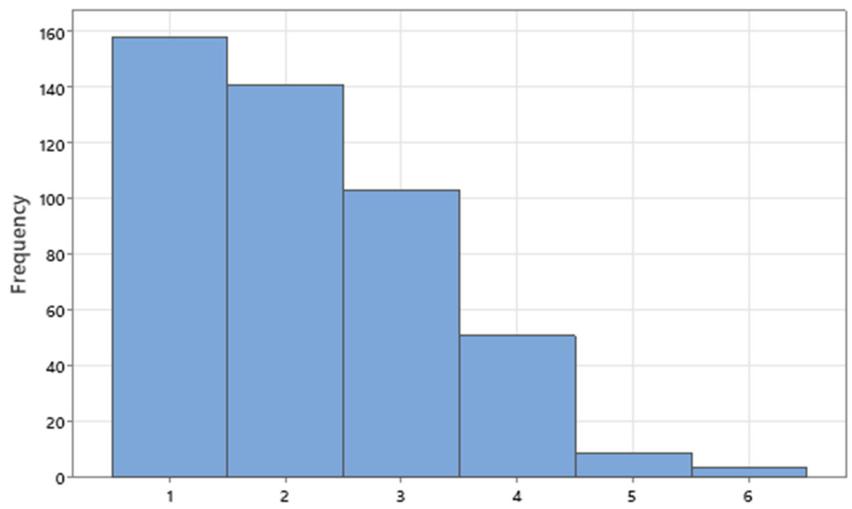

Figure S1: Physical Functioning dimension.

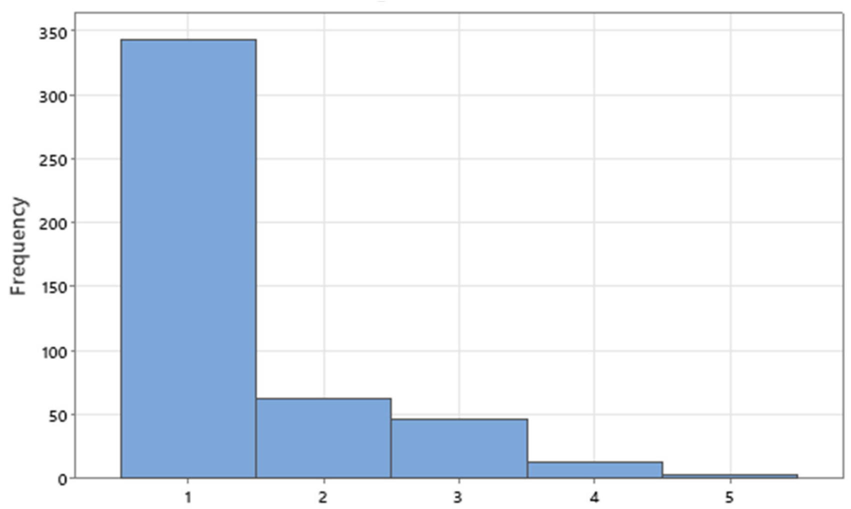

Figure S2: Social Functioning dimension.

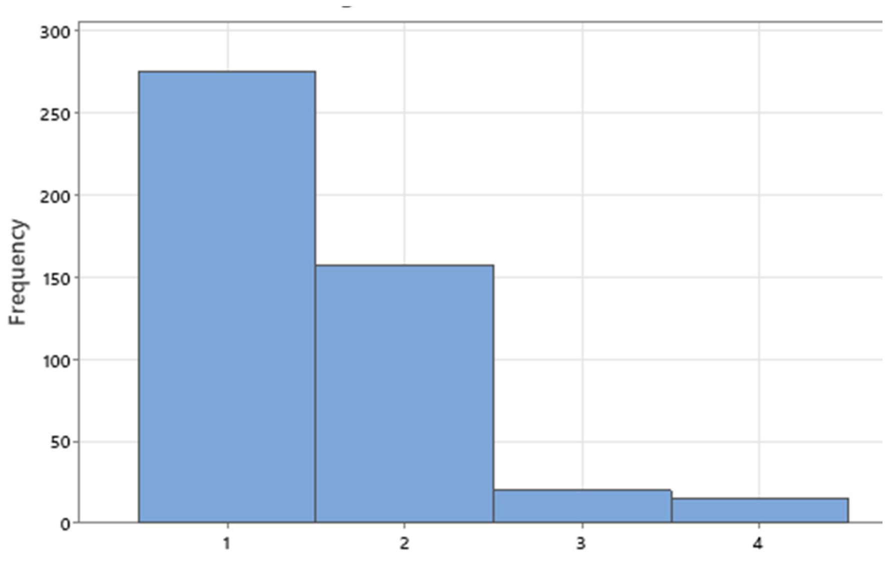

Figure 3: Role limitations dimension

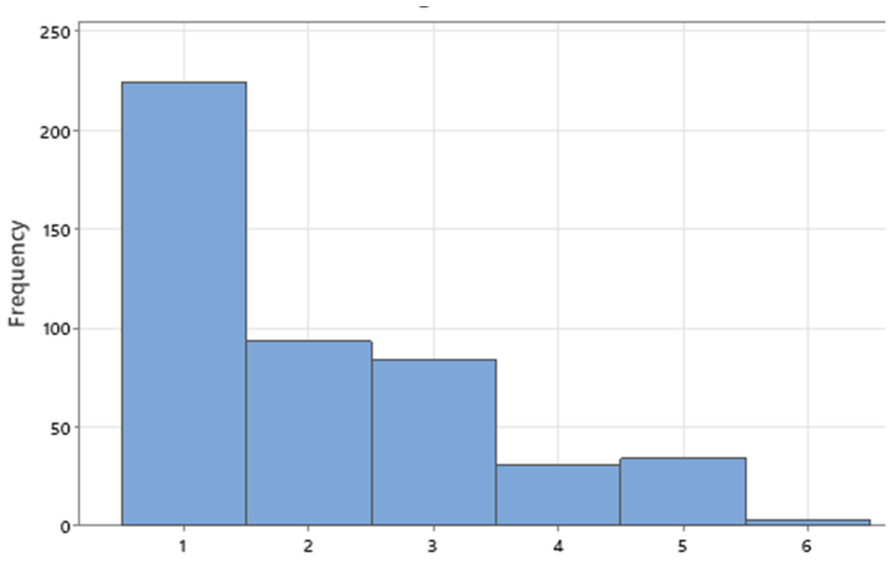

Figure 4: Pain dimension

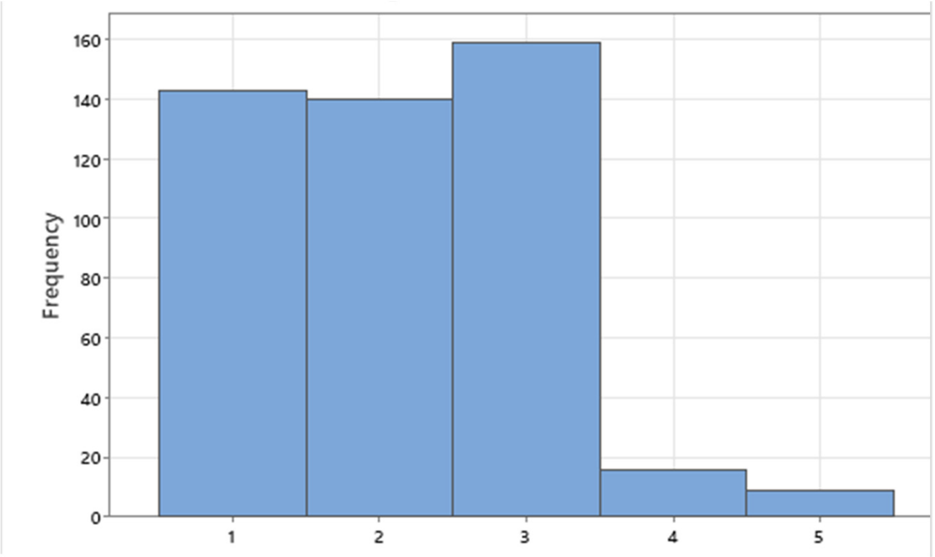

Figure 5: Mental Health Dimension

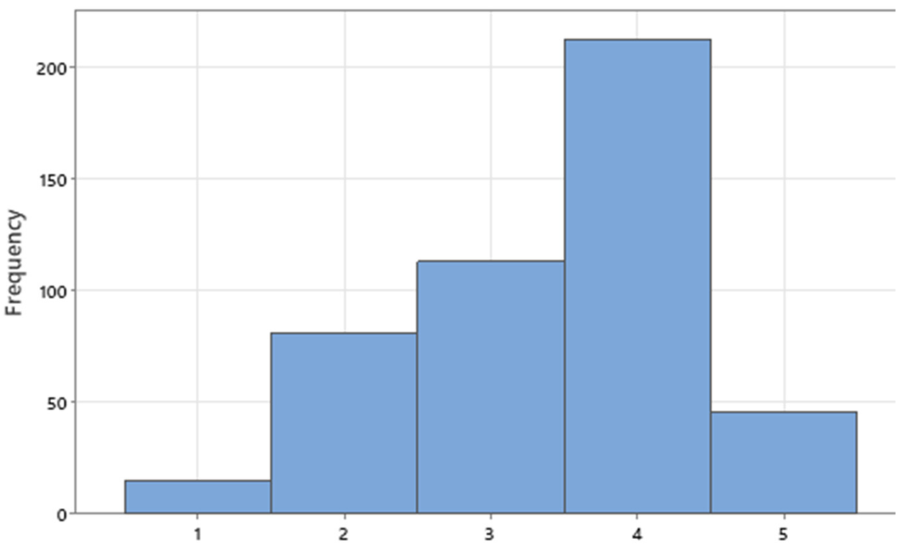

Figure 6: Vitality Dimension

Histograms for the six elements of SF-6D at baseline are shown above. For the dimensions of; Physical Functioning, Role Limitation, Pain, Mental Health and Social Functioning (Figures 1 to 5) lower values represent healthier outcomes and the skewed nature of the histograms to the left demonstrates participant's high rating of wellbeing across the dimensions at baseline. For the Vitality dimension (figure 6) higher vitality ratings are represented by higher values on the scale as this dimension is reversed scored. The skewness to the left on this histogram again represents participant's higher ratings of vitality at baseline
